# Optimizing Dietary Restriction for Genetic Epistasis Analysis and Gene Discovery in *C. elegans*

**DOI:** 10.1371/journal.pone.0004535

**Published:** 2009-02-20

**Authors:** William Mair, Siler H. Panowski, Reuben J. Shaw, Andrew Dillin

**Affiliations:** 1 Molecular and Cell Biology Laboratory, The Salk Institute for Biological Studies, La Jolla, California, United States of America; 2 The Howard Hughes Medical Institute, La Jolla, California, United States of America; University of Minnesota, United States of America

## Abstract

Dietary restriction (DR) increases mammalian lifespan and decreases susceptibility to many age-related diseases. Lifespan extension due to DR is conserved across a wide range of species. Recent research has focused upon genetically tractable model organisms such as *C. elegans* to uncover the genetic mechanisms that regulate the response to DR, in the hope that this information will provide insight into the mammalian response and yield potential therapeutic targets. However, no consensus exists as to the best protocol to apply DR to *C. elegans* and potential key regulators of DR are protocol-specific. Here we define a DR method that better fulfills criteria required for an invertebrate DR protocol to mirror mammalian studies. The food intake that maximizes longevity varies for different genotypes and informative epistasis analysis with another intervention is only achievable at this ‘optimal DR’ level. Importantly therefore, the degree of restriction imposed using our method can easily be adjusted to determine the genotype-specific optimum DR level. We used this protocol to test two previously identified master regulators of DR in the worm. In contrast to previous reports, we find that DR can robustly extend the lifespan of worms lacking the AMP-activated protein kinase catalytic subunit AAK2 or the histone deacetylase SIR-2.1, highlighting the importance of first optimizing DR to identify universal regulators of DR mediated longevity.

## Introduction

Limiting food intake to approximately 60% of the amount an organism eats given *ad libitum* access extends lifespan in a variety of species [1]. Understanding the mechanisms underlying this phenomenon is of medical interest because of the impact DR has on age-related pathology in mammals; DR has been shown to delay the onset and reduce the severity of several diseases including, but not limited to diabetes, auto-immune disease, and many forms of cancer [2]. That organisms can alter their longevity in response to changes in diet is thought to be an evolutionary adaptation to survive periods of low food availability in the wild [3]. During times of famine the survival rate of an organism's offspring would be diminished. Under these circumstances, the adaptive strategy is to shut down or greatly reduce reproduction and redirect the limited resources available towards somatic maintenance to increase the chances of survival until food is plentiful [4]. In accordance with this idea, DR not only increases lifespan but also reduces fecundity [5]–[7]. Furthermore, subsequently re-fed DR animals can reproduce at advanced ages when chronically control-fed animals are no longer reproductive [8].

If this evolutionary theory is correct and the existence of a DR effect in diverse organisms is adaptive, the genetic mechanisms regulating this lifespan extension might be conserved between species. Using genetically tractable, short-lived model organisms rather than rodent models to study DR therefore becomes appealing and may lead to the identity of conserved genetic pathways required for increased longevity in response to DR [9]. Furthermore, understanding which genetic pathways regulate the response to DR might facilitate the design of targeted therapeutic compounds that separate the beneficial effects of DR on health from its detrimental effects; although DR increases lifespan and resistance to many age-related diseases it can also have a negative impact on libido, stamina, wound healing ability and cold tolerance [10]. Maintaining a low food intake also imposes a psychological challenge that would be negated by DR mimetics [10].

Over the last decade there has been an increase in the study of DR in genetically tractable model organisms, in particular *S. cerevisiae* [11], *C. elegans* [12] and *D. melanogaster* [13] resulting in a variety of key nutrient responsive proteins being implicated in the DR pathway. These include but are not limited to components of the insulin/IGF-like growth factor pathway (IIS), the sirtuins, AMP-activated protein kinase (AMPK) and the target of rapamycin (TOR) and their role in DR in different species has been reviewed comprehensively elsewhere [9], [11]. Of particular interest are proteins suggested to be ‘master regulators’ of DR; proteins upon whose presence the DR longevity response is dependant and that therefore lie upstream of the causal mechanisms for the DR effect.

Reduction in food intake is likely to impact upon a host of nutrient sensing and metabolism-related pathways and as such it seems counter-intuitive that the physiological changes induced by DR should converge upon one single factor. Despite this, there exists in the literature many reported examples of such regulators of DR. These include the histone deacetylase Sir2p [14]–[16], the protein kinase AMPK [17], the serine/threonine protein kinase TOR [18], [19] and histone deacetylase Rpd-3 [20], along with the transcription factors PHA-4 [21], SKN-1 [22] and DAF-16 [17]. However, along with the increase in the study of DR on lower organisms has come debate upon the correct DR protocol to use for different species [23]. Further to this, deletion of specific master regulators of DR seems to block lifespan extension in response to certain DR protocols but not to others [17], [24]. This leads to two possible explanations; 1) Different DR protocols impact upon different pathways, each of which are dependent upon specific regulators or 2) sub-optimal DR regimes can result in false-positive identification of potential master regulators.

With the onset of the comparative ‘omics’ era, the need to identify which of these two explanations is the case becomes paramount, since much time and research money is invested in mammalian aging research based upon initial findings taken from lower organisms. If different DR protocols in the same species function through distinct regulators, the likelihood that conserved pathways regulate the DR response across the evolutionary ladder perhaps becomes diminished. Alternatively, if using differing and sub-optimal DR protocols can result in false identification of DR regulators, it is important to develop a unified approach that minimizes such occurrences.

Classical genetic epistasis analysis reasons that if two interventions result in an additive phenotypic response they lie in separate pathways. However, this rule does not hold if the phenotype from either intervention is not maximized. For example, weak hypomorphic mutations in the insulin/IGF-like receptor *daf-2* extend worm lifespan, yet this lifespan extension in further enhanced by *daf-2* RNAi [25], [26]. In this case both interventions clearly lie in the same pathway despite there being an additive response when both are applied together.

**Figure 1:**
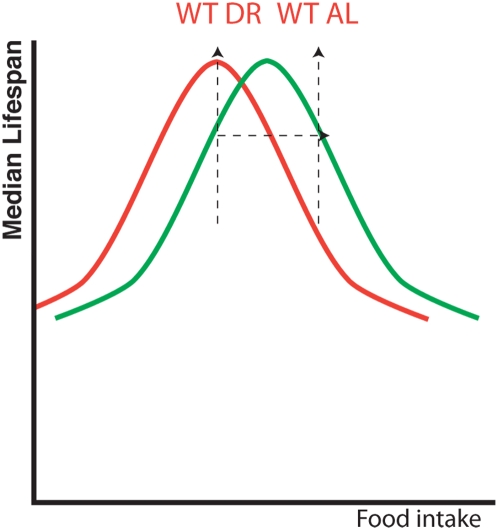
The importance of optimizing DR for different genotypes before perfoming epistasis analysis. Testing only 2 food intake levels can lead to false identification of master regulators of DR. Schematic model of lifespan of wild type animals shows a parabolic response to food intake (red line). If a genetic mutation alters the response of an animal to DR, the position of this curve on the x axis can be shifted (e.g. green line) [27]. In this situation, using only two food levels based on the wild type's *ad libitum* (WT AL) and dietary restriction (WT DR) position would falsely suggest DR does not increase longevity of the mutant despite the fact it shows a clear lifespan extension when food intake is reduced (green line). Testing a range of food intake levels rather than just two would resolve the green line, revealing a response to DR not seen if only 2 levels were used.

To informatively interpret data from classical epistasis analyses testing two interventions that affect longevity, lifespan from one intervention must therefore be maximized before another is added [27]. This is especially important when investigating the effect of genetic mutations on environmental perturbations such as DR because lifespan extension by DR is not binary but instead a graded response. For example, as food intake is reduced from *ad libitum* levels, lifespan gradually increases until a food level that maximizes longevity is reached, past which further reduction in food intake begins to shorten lifespan as animals enter starvation. This parabolic response of average lifespan to food intake has been observed in yeast [28], worms [29], flies [27] and mammals [30]. By its very nature, the parabolic curve indicates that the same lifespan can result from more than one specific nutritional intake level (Figure 1). Furthermore, different genotypes can respond to DR differentially such that the food intake that maximizes lifespan for one genotype may not be the same as that which maximizes the lifespan of another (Figure 1) [27], [31], [32]. Indeed, disruption to genetic pathways involved in nutrient sensing/signaling is likely to shift this optimal food intake level as the organism is effectively partially dietarily restricted by its genotype [9]. It is therefore paramount that DR is first ‘optimized’ for each genotype tested before epistasis analysis with a second genetic or pharmacological intervention is performed, i.e. the animals must be subjected to a range of food intake levels and that which causes maximum lifespan is set as the DR level for that genotype and used for epistasis studies.

If lifespan extension due to DR is dependent upon a single factor, removal of that factor will completely abolish the DR effect. Rather than a parabolic response of longevity to DR there will be no interaction between food intake and lifespan when the DR factor is removed. Longevity will be the same at all food intake levels and plotting average lifespan against food intake would produce a horizontal line across the DR range. Crucially however, testing this hypothesis requires that lifespan be measured across a range of food intake levels (Figure 1). This technique has been used in two recent studies in *C. elegans* to show that two transcription factors, SKN-1 and PHA-4 are both necessary for a dietary restriction response [21], [22]. In both cases, worms lacking either transcription factor showed the same lifespan across a range of dietary intake levels ranging from near starvation to *ad libitum*.

In separate studies in the worm, deletion of the gene encoding AAK2, the catalytic subunit of AMP-activated protein kinase, or the gene encoding the histone deacetylase SIR2.1, was reported to block lifespan extension by DR [15], [17]. However, in these studies lifespan of the mutant animals was only tested at two food levels; a control level and then either DR imposed by one food dilution or mutation in the gene *eat-2* (which results in decreased food intake [33], [34]) respectively, i.e. the DR protocol was not first optimized for the mutant.

Here we expand upon a protocol for applying DR to *C. elegans* [6] that meets criteria required for an invertebrate method to be comparable to mammalian DR. Using this method we show that there is no compensation for food intake under DR conditions and that reproductive rate correlates with feeding. We then use this method to test whether mutations in *aak-2* or *sir-2.1* attenuate the lifespan extension seen in *C. elegans* when they are subjected to decreased concentrations of bacterial solution as a food source. Unlike previously published results, mutations in either *aak-2* or *sir-2.1* fail to block lifespan extension by DR using our protocol, which robustly extends lifespan of wild type worms whilst reducing both food intake and reproduction. These results indicate the importance of genotype-specific optimization of DR protocols if we are to obtain consistent results across different species and between different laboratories, and provide an easy DR method to be adopted as the standard for DR studies in the worm.

## Results and Discussion

### Defining a DR protocol for epistasis analysis in C. elegans

DR studies in the nematode *C. elegans* were first carried out by Klass in 1977 [6], yet still no consensus exists as to the best methodology to apply DR to worms that is consistent with mammalian DR regimes, and several different methods are used by different laboratories: Klass bacterial dilution [6], Dillin laboratory bacterial dilution [21], Guarente laboratory bacterial dilution [22], Brunet laboratory bacterial dilution [17], Vanfleteren laboratory bacterial dilution [29], genetic DR surrogate by *eat-2* mutation [33], peptone reduction [35], axenic media [36], complete removal of food [37], [38] and chemical inhibitors of glycolysis [39], along with unpublished protocols such as every other day feeding and sugar dilution (Table 1). Comparing results between laboratories becomes problematic with such a diversity of protocols and DR research needs a single defined approach that could be used by the *C. elegans* community as a whole to compare with vertebrate studies. Alternatively, for each gene thought to play a role in DR, every protocol must be tested and new protocols rigorously analyzed for their effect on feeding, behavior and reproductive functions, all hallmarks of mammalian DR.

**Table 1:** Comparison of different DR protocols for *C. elegans*.

Method	Average lifespan of DR group (days)	Lifespan extension	Reduces Reproduction?	Optimizable for different genotypes?	Track individual worm lifespan?	Reduced food intake	Ref.
Klass BDR	25.9*	72.6*	Yes	Yes	Yes	NT	[6]
Vanfleteren BDR	12**	140%**	NT	Yes	No	NT	[29]
Dillin BDR	42.0	82.6%	Yes	Yes	Yes	Yes	[21]
Guarente BDR	32.8	27.6%	Yes	Yes	Yes	NT	[22]
Brunet BDR	23.55	18.4%	NT	No	Yes	Yes	[17]
Kennedy DD	30	50%	NT***	No	Yes	Yes	[37]
Zou DD	21.8	41.4%	NT***	No	Yes	Yes	[38]
*eat-2 (ad1116)*	30.6	57%	Yes	No	Yes	Yes	[34]
Peptone reduction	18.2	32.8%	No	Yes	Yes	No	[35]
Axenic media	25.9	79.9%	NT	No	Yes	No	[36]
Glycolysis inhibition	20.6	16.4%	NT	Yes	Yes	No	[39]

* BDR implemented during larval growth.

** inferred from graph.

*** Cannot be tested as food deprivation causes *C. elegans* to withhold eggs, which eventually hatch internally.

NT = Not tested.

To translate findings of DR in the worm to other systems it is important that the protocol used not only increases lifespan, but also re-capitulates other phenotypes of DR seen in mammalian systems. Criteria for such a protocol are that 1) DR animals have reduced reproductive fitness despite longer lifespans [7], 2) DR does not extend lifespan by reducing a husbandry-specific toxicity associated with the food source for that particular organism [12], 3) Food intake is reduced, not just food availability [23]. Furthermore, to be used for informative epistasis analysis, invertebrate DR protocols must also allow tracking of individual lifespans and be optimizable for different genotypes [27]. We tested whether the protocol used in our lab, bacterial dilution DR (BDR), met these criteria.

BDR involves transferring reproductive, young, adult worms to liquid bacterial cultures of either high (*ad libitum*) or low (BDR) concentrations. To avoid detrimental effects of DR on development, worms are only placed on the BDR regime after they reach adulthood on standard worm husbandry bacterial plates [40]. Furthermore, BDR is not a batch culture system of large numbers of worms in liquid culture, but instead uses only 15 worms per/ml food culture, and worms are moved to fresh media twice weekly. The bacterial cultures are non-proliferative to maintain constant concentrations at all times (see methods section for more details).

### 1) BDR reduces reproduction

One key signature of mammalian DR is that the reduced food-intake regime increases lifespan yet decreases reproduction, such that the dietary level that optimizes these two life history traits is separable. This finding explains evolutionary theory often used to explain the DR response; when food is plentiful the strategy that will maximize fitness is boom and bust [4], i.e. invest in reproduction even at the cost of somatic maintenance because extrinsic factors (predation, disease etc) will likely cause death long before old age in the wild. In times of famine however, offspring are unlikely to survive, therefore investing what limited resources are available into self-preservation and hazard avoidance (for example, disease resistance, youthfulness and mobility) becomes a better strategy in the hope that, when food returns, the individual is still alive to reinitiate reproduction.

**Figure 2:**
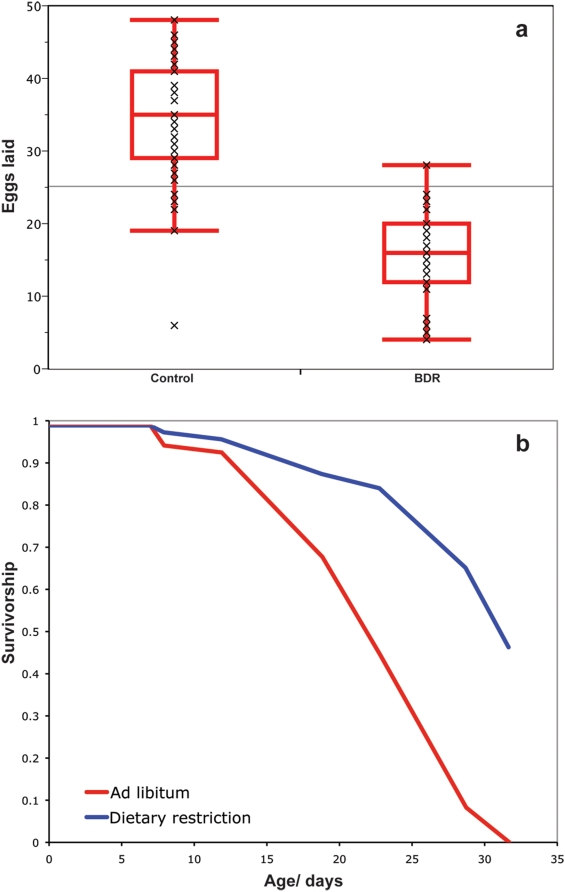
BDR and reproduction. a. BDR significantly reduces the rate of egg-production of wild type *C. elegans*. Median egg production in 7 hours; Control feeding = 35, BDR = 16, (Non-parametric Wilcoxon test, P<0.0001). b. Lifespan of male wild type *C. elegans* on control and BDR feeding regimes. BDR significantly extends the lifespan of male worms. Median Lifespan; Control = 23 days, BDR = 32 days. 39.1% extension (Log rank test, P<0.0001).

Similar to mammalian DR, BDR significantly reduces the rate of reproduction, as measured by egg production by hermaphrodites, compared to controls (Median eggs laid per adult worm in 7 hours: Control = 35, BDR = 16, Non-parametric Wilcoxon test, P<0.0001, Figure 2a). However, as is the case for DR in mammals and fruit flies [1], [41], lifespan extension under BDR is not the direct result of reduced egg production; BDR increases the lifespan of both wild type male *C. elegans* (Log rank test, P<0.0001. Figure 2b), and sterile normal lived hermaphrodite *glp-4* mutants (data not shown).

### 2) Species-specific effects on lifespan

Several species-specific effects need to be considered when designing a DR protocol for *C. elegans*, including differential levels of internal invasion by proliferative bacteria and varying oxygen availability in control and DR conditions. Worms suffer increased bacterial invasion of their tissue with age [42] and preventing this invasion by culturing *C. elegans* on non-dividing bacterial lawns increases lifespan [43]. Raising worms in diluted liquid bacterial cultures may therefore increase lifespan by reducing this toxic effect. However, the bacteria used in our BDR protocol are non-dividing due to the presence of antibiotics (bactericides and bacteriostatics) in the solution. Growing *C. elegans* on lawns of dividing bacteria seeded at differing concentrations [17] may well result in reduction of both food intake and also bacterial invasion and as such at least part of the life enhancing mechanism invoked by this DR protocol may be specific to *C. elegans*.

*C. elegans* in batch culture show very short lifespans, which may be due to hypoxia when the worm population concentration is dense [29]. Our BDR husbandry method has only 15 worms per ml of liquid and is not batch culture – worms are moved to new media twice weekly. Furthermore, oxygen saturation is >95% that of air at all food concentrations (Table S1), ruling out hypoxia as the cause of lifespan shortening at control food levels.

If high food intake causes toxicity in the worm we would predict that increased feeding would decrease both lifespan and reproduction as the worms ingested increasing amounts of the toxin. This is the case for one worm DR protocol, reduction of peptone levels in worm media [35], suggesting that it is the dilution of the toxic effects of peptone rather than DR that increases lifespan in this case. Since control worms using our bacterial media reproduce more than their BDR counterparts, it seems unlikely they are suffering from increased stress from either a toxin in the media or the presence of non-dividing bacteria.

### 3. BDR decreases food intake

As with protocols for *Drosophila* [13], most DR techniques used for *C. elegans* reduce the nutritional quality rather than the absolute quantity of the food, with animals typically given *ad libitum* access to foods with varying concentrations of nutrients. It is therefore feasible that when presented with a more diluted food source worms compensate by increasing their feeding rates and as such do not have decreased food intake under DR. Although the reduced reproductive rates using our BDR method suggest the worms are food limited we wanted to confirm this both directly by measuring food intake on different nutritional regimes and indirectly, by measuring developmental rate under BDR feeding.

**Figure 3:**
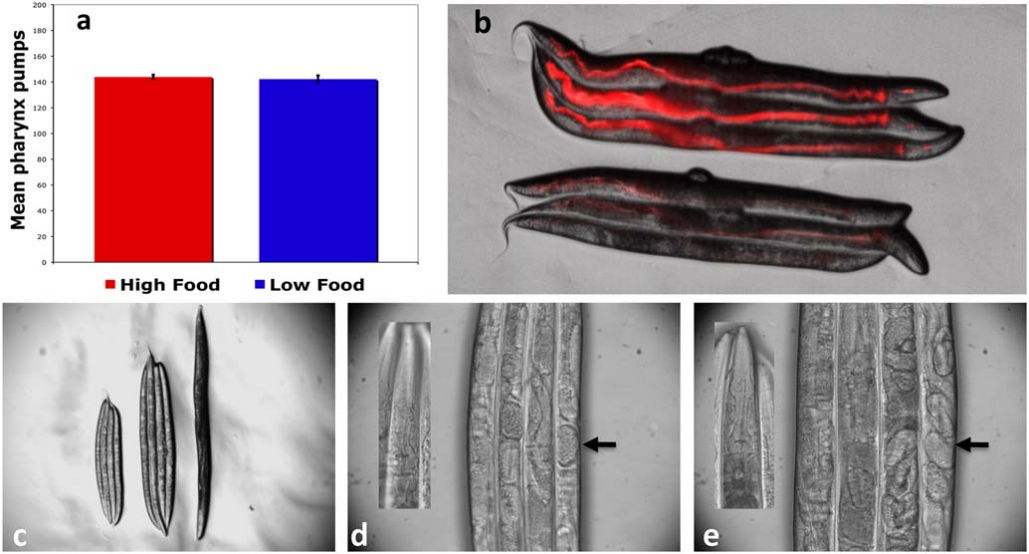
Worms do not compensatory feed under BDR conditions. a. Pharyngeal pumping rates under control feeding and BDR are not significantly different (Mean pumps/30 seconds; Control = 143.9, BDR = 142.5. Non-parametric Wilcoxon test, P = 0.946). b. BDR decreases food intake. Wild type worms were fed tdTOMATO expressing *E. coli* for 24 hours at control (top 3 worms) and BDR (bottom 3 worms) concentration. c. L2 larval stage wild type worms grown on a BDR regime (left 3) are smaller than those grown in control liquid medium (center 3) and therefore do not compensatory feed. Worms grown in liquid media are smaller than those grown on *E. coli* lawns on plates (right). d & e. Liquid culture does not cause growth arrest. L2 larval stage wild type worms grown in either BDR (d) or control (e) media reach adulthood as shown by the presence of mature oocytes (arrows) and the lack of a dauer-specific oral plug (inset).

Pharyngeal pumping rates are used as an indicator of feeding rates in *C. elegans* [44] and were not significantly different at the bacterial concentration that maximizes lifespan of wild type worms compared to control food medium (Non-parametric Wilcoxon test, P = 0.946, Figure 3a). In accordance with this, direct measurements of food intake confirmed worms do not exhibit compensatory feeding rates and food intake is lower at the BDR concentration. Ingestion of fluorescent bacteria expressing tdTOMATO was significantly lower than controls after 24 hours of BDR in two replicated trials (Mean pixel intensity: control experiment 1 = 5.82 (SD = 2.12), BDR experiment 1 = 1.65 (SD = 0.49), control experiment 2 = 5.60 (SD = 4.10), BDR experiment 2 = 1.11 (SD = 1.13). Both repeats: P<0.0001, Students t Test. Figure 3b).

Examination of growth rates in control and BDR bacterial concentration food media further supported the finding that worms cannot compensatory feed when given a dilute food source. Eggs hatched in M9 buffer without a food source arrest at the L1 larval stage and these arrested larvae do not resume normal growth when placed in the BDR solution (0.15 OD), yet do exit arrest and reach adulthood when placed at control bacterial concentrations (1.5 OD) (data not shown). In contrast, *eat-2* L1 arrested mutant larvae do not exit larval arrest even at control bacterial concentrations (1.5 OD), further supporting their reduced food intake relative to wild type and their inability to compensatory feed (data not shown).

Synchronized L2 larval stage wild type worms moved to BDR liquid food took 24 hours longer to become gravid adults at 20 degrees centigrade and are 27.9% smaller than control fed worms (P<0.0001, students t-test, Figure 3c). However, this size reduction is not due to dauer or larval arrest but rather worms grown in BDR culture become small reproductive adults (Figure 3 d & e); they do not have the dauer-specific oral plug (Figure 3d inset) and examination under a light microscope shows them to be gravid (Figure 3d). If *C. elegans* increase feeding rates in the BDR liquid to compensate for reduced food quality we would have expected animals not to arrest development or show reduced growth.

### Comparison of BDR to other worm DR protocols

**Figure 4:**
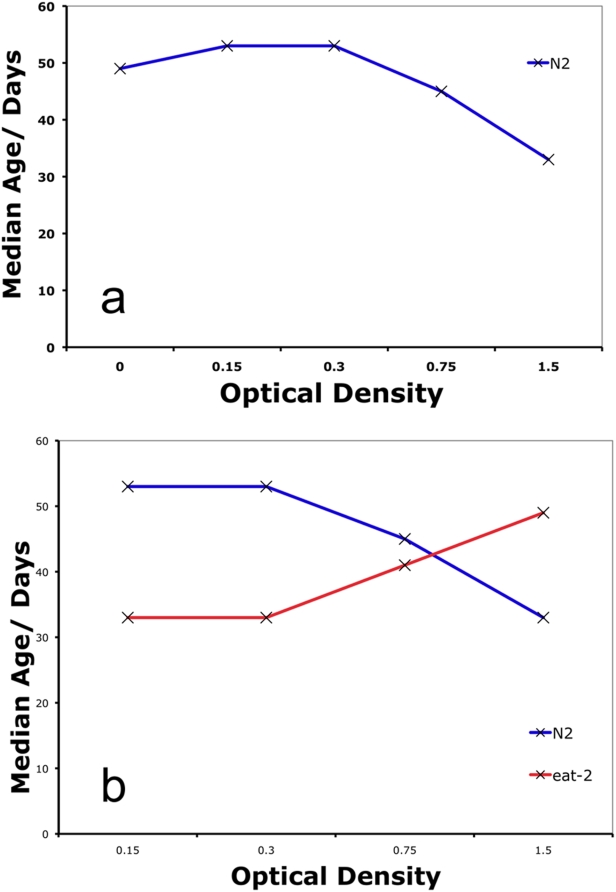
BDR robustly extends lifespan. a. Median lifespan of wild type worms across a range of food concentrations. Median lifespan increases as food concentration is reduced from an optical density (OD) of 1.5 and peaks at an OD of 0.15 and 0.3. b. *eat-2* mutant worms live longer than wild type worms at a high food concentration (1.5 OD) but are short lived at lower concentrations (0.15&0.3 OD). Log rank test, P<0.0001 for all comparisons of *eat-2* versus controls.

Adult worms cultured in the BDR method show the expected parabolic response of lifespan to changes in the concentration of the bacterial food source (Figure 4a), with lifespan peaking at an OD of 0.15. The percent lifespan extension seen using BDR (80–100%) (Table 1) is greater than many other worm DR protocols used, with one exception being BDR in high worm concentration batch culture [29]. However, the average lifespan of worms using this protocol is only 5 days (*ad libitum*) and 12 days (DR) suggesting that something other than food intake is limiting lifespan under these culturing conditions. In contrast, worms grown on BDR have an average lifespan of over 50 days (Figure 4a), longer than any other reported method (Table 1).

Since BDR therefore reduces reproduction and feeding rates while robustly extending lifespan, it is a method of dietary restriction that successfully recapitulates many aspects of mammalian DR (Table 1). Crucially, an advantage of BDR over many other worm DR protocols is that it is optimizable, such that it can be imposed at varying levels of severity and a food intake level that generates maximum lifespan for any genotype can be determined. This is not the case for Dietary Deprivation (DD), another worm DR protocol where worms are grown on bacterial lawns and then moved onto agar plates without a food source [37], [38]. Lifespan extension by DD was suggested to be a *bona fide* DR protocol since it is not additive with the increased longevity caused by mutations in *eat-2* [37], [38]. However, this would be expected since pharyngeal pumping defects possessed by *eat* mutants cannot reduce intake when no food is present; even if *eat-2* extended lifespan through separate mechanisms to DD it could not have an effect when no food is available.

We observed a similar result to DD using BDR - worms are longer lived when transferred as adults to an S-Basal media containing no bacteria than media with high bacterial concentrations (Figure 4a). Importantly however, a bacterial concentration of zero was not the optimum for lifespan of wild type worms (Figure 4a) suggesting that *C. elegans* given no food as adults are under starvation stress. This is in disagreement with experiments testing the effect of a range of bacterial concentrations on worm lifespan using a plate-assay, where no food caused the maximum lifespan extension [37]. It may be that this study did not test a fine enough range of bacterial dilutions and therefore missed the optimal food intake for wild type worms. Certainly, in the quest to find protocols for DR in worms that mimic those used for mammalian studies, complete removal of food does not fulfill the criterion of ‘under nutrition without malnutrition’ [1] and may in part invoke worm-specific starvation responses that may or may not translate to the vertebrate DR paradigm.

Epistasis analysis testing the interaction between genetic mutations and DR in the worm is usually carried out using *eat-2* mutant worm strains [45]. *eat-2* encodes a ligand-gated ion channel subunit that functions in the pharynx to regulate the rate of pharyngeal pumping [33]. Compared to wild type, *eat-2(ad1116)* mutant animals have reduced pharyngeal pumping rates and are long-lived. They have therefore been used as a genetic surrogate of dietary restriction [33], [34]. If the pumping rate decline of *eat-2* mutants results in dietary restriction, one would predict that they would starve at a higher bacterial concentration than wild type animals. We tested this by culturing *eat-2(ad1116)* mutants in liquid bacteria using the BDR method. *eat-2(ad1116)* mutants are long-lived compared to controls at high food concentrations (Log rank test, P<0.0001 and Figure 4b), yet are short lived when cultured at the bacterial concentration that maximizes the lifespan of controls (Log rank test, P<0.0001 and Figure 4b). This supports the model that *eat-2(ad1116)* mutants are in a dietarily restricted state. As such, *eat-2* is a *bone fide* DR method but one that represents just one point on the food continuum. Using *eat-2* as a DR model is also complicated by the fact that the animals are food-limited during development and as such have delayed growth rates compared to controls [33].

Although use of the *eat-2* mutant animals as a DR surrogate is less labor intensive than BDR it cannot be optimized in the same way and the lifespan extension seen in the same *eat-2* mutant varies between labs and between experimenters. Factors that govern this effect may include thickness of the bacterial lawn on the plate, culture temperature and worm husbandry. Differences in these may explain cases where epistasis experiments using *eat-2* show divergent results between laboratories [18], [46]. Different labs reporting different results using the same *eat-2* mutants strain [18], [46] highlights the difficulty of performing epistasis analysis on a non-optimizable DR method such as *eat-2* mutation.

Only a DR method that can be optimized for different genotypes, as is the case for BDR can give informative data for use in epistasis analysis with a second intervention [27]. We therefore went on to re-evaluate if AMPK and SIR-2.1, both previously reported to be master regulators of DR using protocols that had not been specifically optimized for the mutant strain, were required for lifespan extension by BDR.

### AMPK is not required for lifespan extension by BDR

AMPK is activated under low energy conditions (high AMP∶ATP ratio) and is suggested to act as a nutrient sensing switch [47], turning off energy consuming processes such as protein synthesis through the inhibition of TOR signaling [48], [49]. AMPK regulates many critical metabolic functions in mammals, acting in the hypothalamus to promote food intake [50] and regulating glucose homeostasis [51], [52]. Similar to mammals [47], [53], *C. elegans* have two catalytic subunits of AMPK, *aak-1* and *aak-2* [54]. AMPK plays a role in aging and stress resistance in the worm since lifespan extension via reduced insulin/IGF signaling is largely abolished in worms mutant for the AMPK alpha subunit (AAK2), whilst over-expression of *aak-2* increases longevity [54]. In *C. elegans*, AMPK is required for the extreme longevity seen in dauer larvae, an alternate spore-like developmental stage induced by low food availability or high population density [55]. AMPK is therefore an appealing candidate as a regulator of DR-induced lifespan extension and indeed strains homozygous for the *aak-2(ok524*) loss-of-function deletion do not show lifespan extension using a solid plate method of DR [17].

**Figure 5:**
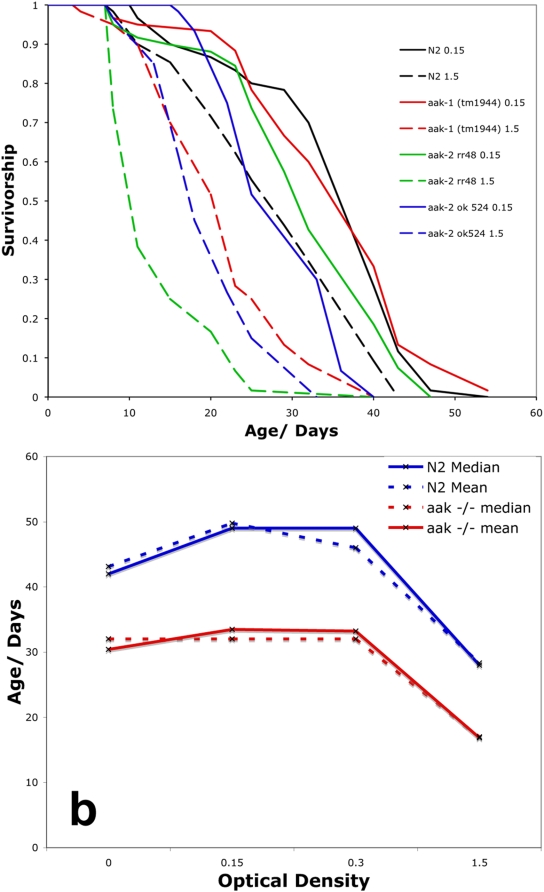
AMPK is not required for lifespan extension by BDR. a. BDR (0.15 OD, solid lines) increases the lifespan of wild type (black), *aak-1(tm1944)* mutants (red), *aak-2(rr48)* mutants (green) and *aak-2(ok524)* mutants (blue) compared to control feeding (1.5 OD, dashed lines). Log rank test, P<0.0001 in all cases. b. Median (solid) and mean (dashed) lifespan of wild type (blue) and *aak-1; aak-2* double mutants (red) across a range of food concentrations. *aak-1; aak-2* double mutants are shorter lived than wild type at all food intake levels (Log rank test, P<0.0001 in all cases). *aak-1; aak-2* double mutant lifespan is significantly increased by BDR. Median lifespan of *aak-1; aak-2* double mutants; Control food level (1.5 OD) = 17 days, BDR (0.15 OD) = 32 days. Log rank test, P<0.0001.

However, unlike the previous study [17], we found clear lifespan extension via BDR for worms homozygous for the same *aak-2(ok524)* mutant allele using the BDR concentration that maximizes lifespan of wild type worms (Figure 5a, Log rank test, P<0.0001). Lifespan extension by BDR was also seen in worms homozygous for another *aak-2* loss-of-function mutation, *aak-2(rr48)*. BDR also increased longevity of *aak-1(tm1944)* homozygotes, which carry a deletion in the gene encoding the second AMPK catalytic subunit AAK-1 (Figure 5a).

In mammals there is redundancy between the two alpha catalytic subunits of AMPK [56]. To test if this was the case in worms we measured the effect of BDR on *aak-1(tm1944) III; aak-2(ok524) X* double mutants (Figure S1a). These worms showed no phospho-AMPK activity as determined by AMPK phospho-specific antibody western blot analysis (Figure S1b). Under standard plate assays the *aak-1(tm1944) III ; aak-2(ok524) X* double deletion mutant had slightly delayed development, reduced reproduction and shorter lifespan compared to wild type (mean wildtype = 16.4 days, mean AMPK null = 13.5 days, p<0.0001, Log Rank Test).

Despite a lack of AMPK, *aak-1(tm1944) III ; aak-2(ok524) X* double mutant worms showed a significant extension of lifespan by dietary restriction from BDR (Figure 5b, Log rank test, P<0.0001). Therefore AMPK is not required for lifespan extension by BDR in *C. elegans*. AMPK is required for development in mammals [56] and *Drosophila* [*57*], therefore the fact that *aak-1(tm1944) III ; aak-2(ok524) X C. elegans* double mutants can become viable adults suggests that there may be compensation by another AMPK-family kinase in these worms.

### Neither Sir-2.1 nor Sir-2.3 are required for lifespan extension by BDR

**Figure 6:**
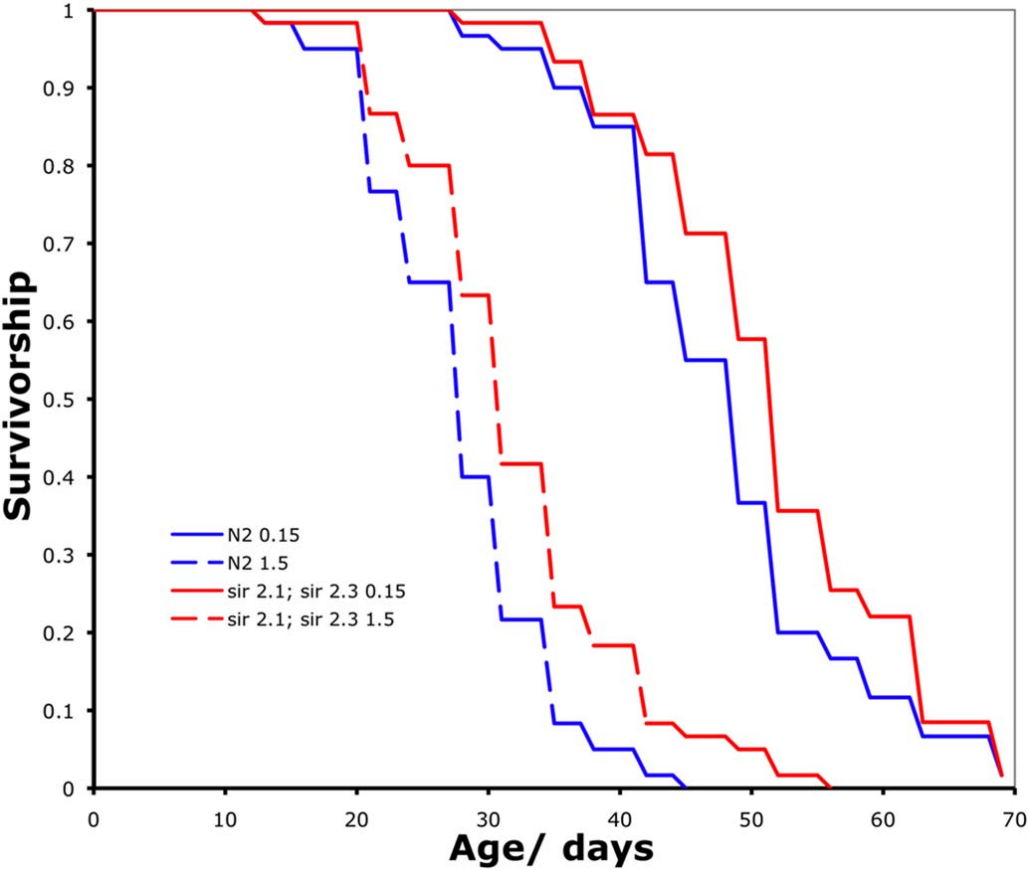
BDR increases the lifespan of *sir-2.1; sir-2.3* double mutants worms. Wild type (blue) and *sir-2.1; sir-2.3* mutants (red) are significantly longer lived under BDR (solid lines) compared to control (dashed lines) feeding (Log rank test, P<0.0001 in both cases).

Sir2p is a NAD dependent histone deacetylase that has been reported as being necessary for lifespan extension via DR in budding yeast [14], although controversy about its role as a master regulator of DR in yeast remains [9]. More recently DR was shown to extend lifespan in the filamentous fungus *Podospora anserina* in a *PaSir2* independent manner [58]. SIR-2.1 is the closest worm homologue to the yeast Sir2p and, similarly, was shown to be required for *eat-2* mediated DR in *C. elegans* [15]. As with the work in yeast and *P. anserina* however, there are conflicting reports suggesting that DR can extend the lifespan of *sir-2.1* mutants [18], [37], [59]. There are four sirtuins in the worm, opening up the potential for redundancy in their role in mediating the effects of dietary restriction. We therefore examined the effect of BDR on *sir-2.1(ok434) IV; sir-2.3(ok444) X* double mutant worms. However, BDR robustly extended the lifespan of the double homozygous *sir-2.1(ok434) IV; sir-2.3(ok444) X* mutants (Figure 6, Log rank test, P<0.0001). Therefore neither SIR2.1 nor SIR2.3 are necessary for BDR mediated lifespan extension in the worm.

*sir-2.2* and *sir-2.3* are closely linked in the *C. elegans* genome making construction of a *sir-2.1; sir-2.2 ; sir-2.3* triple mutant difficult, and we are not aware of any homozygous *sir-2.4* mutant that is viable. Combining RNAi gene knockdown via bacterial feeding and BDR introduces confounding effects of reduced RNAi in the DR group in worms. In the future it will be of great interest to utilize RNAi methods that circumnavigate these technical issues to knockdown all four sirtuins in the worm under *ad libitum* and DR conditions.

### Conclusions

Observing lifespan extension using only one level of dietary restriction in a mutant is enough to show that DR does not depend upon the gene(s) in question, as is the case here for the *sir-2.1(ok434 IV); sir-2.3(ok444) X* double mutant. However, it is important to highlight the difference between lifespan extension by DR being dependent upon a gene versus being independent of it, a distinction that is more than simple semantics. Key nutrient-dependent factors such as AMPK and the sirtuins are likely to be involved in part of the DR response and DR cannot be said to be independent of them if altering their activity changes either the level of DR that optimizes lifespan or the lifespan extension that DR generates. Indeed, changing the activity of either SIR2 or the forkhead transcription factor FOXO, both of which were reported to be required for a DR response in studies using two food levels [15], [17], was subsequently shown to alter the DR response but not block it when a range of DR levels was examined [24], [60]. Therefore DR is not independent of either SIR2 or FOXO, despite neither being required for a DR response.

We have shown that when a robust, optimizable DR method is used, lifespan extension via reduced food intake in the worm does not require either AMPK or the two sirtuins, SIR2.1 and SIR2.3. This is the first example of testing the lifespan of AMPK or sirtuin mutants across a range of food intake levels in the worm and highlights the importance of this strategy if we are to identify potential master regulators of DR. Although both AMPK and the sirtuins may mediate some of the effects of DR in this and other species, neither is responsible for the DR effect in its entirety in *C. elegans*. If only two food intake levels are tested and the lifespan extension seen in wild type animals is weak, the chances of mis-identifying an intervention that alters the DR response as one that blocks it is increased.

BDR represents a DR method that gives robust lifespan extension and avoids many of the pitfalls associated with other worm DR protocols, as discussed above. Like all invertebrate DR methods, BDR does have experimental limitations. The worms are living in liquid throughout their adult life and it is unclear what proportion of their life-history in the wild is spent in similar conditions. However, this does prevent confounding effects of behavior that pose problems to plate based assays, where interventions may be affecting food intake due to the worms spending differing amounts of time within the bacterial lawn. Our method also requires the use of 5-Fluorodeoxyuridine (FUDR) to prevent progeny from hatching and is also more labor intensive than plate based lifespan assays. As with all worm DR methods using bacteria as a food source, combining BDR with RNAi using standard bacterial feeding approaches likely results in a dilution of the DR effect in the BDR cohorts, although this can be circumnavigated by applying alternative RNAi methods.

Despite these caveats, we propose that graded response to food levels by BDR better fulfills the requirements of a worm DR protocol to compare to mammalian DR than other methods currently being used. Crucially, we stress the need to optimize DR for each genotype tested before performing epistasis analysis, and therefore that any alternative to BDR should facilitate such an approach.

In our studies we have attempted to measure additional physiological outcomes of DR in a worm based protocol to ensure as much over lap with mammalian DR as possible. However, until a gene found to be required for longevity in a worm based DR protocol is validated in a bona fide mammalian model of DR, we will not know which worm DR protocol more closely reflects the mammalian condition. Demonstrating such conservation from invertebrates to mammals in the regulation of DR, and in particular showing relevance to human aging and pathology, is the ultimate goal of all aging research on model organisms and one that, if achieved, will facilitate exciting new therapeutic possibilities.

## Methods

### BDR media preparation

*E.coli* (OP50) was grown in 100 ml LB in a 1 L flask O/N at 37°C and spun down at ∼4000 rpm for 10 min. Cells were then washed twice with S-basal/cholesterol/antibiotics solution (1× S-Basal Medium (5.85 g NaCl, 1.0 g K_2_HPO_4_, 6.0 g KH_2_PO_4_, 1.0 ml cholesterol (5 mg/ml in ethanol), Carb (50 µg/ml)+Kan (10 µg/ml)+Tet (1 µg/ml), MQ H_2_0 to one liter – sterile filtered) then re-suspended in S-basal/cholesterol/antibiotics solution and diluted to the required optical density (OD). OD was measured at 600 nm. For experiments with 2 bacterial concentrations, control OD = 1.5, BDR OD = 0.15. When testing a range of dilutions OD = 1.5, 0.75, 0.3, 0.15, 0.

### BDR Lifespan assays

All experiments were carried out in 20°C incubators. Gravid adult worms were placed onto standard nematode growth media [61] plates previously seeded with OP50 bacteria (10 worms a plate). After six hours, adult worms were removed and the eggs allowed to hatch and develop. Enough egg lay plates were prepared for experimental worms assuming approximately 100 eggs per plate. 72 hours after the beginning of the egg-lay, 50 µl of FUDR (100 mg/L M9 solution) was added to each plate to arrest progeny development.

24 hours after addition of FUDR to the plates, worms were transferred using a worm pick to a well of a six well cell-culture plate containing 3 ml of room temperature S-basal/cholesterol/antibiotics solution+FUDR (100 mg/L). The plate was left on slow rotation on a ‘belly-dancer’ shaker in the 20°C incubator for one hour to remove any residual OP50 bacterial clumps stuck to the worms. During this time bacterial solutions for the lifespan (see above) were added to 12-well cell culture plates, 1 ml solution per well+FUDR (100 mg/L), and placed on the 20°C shaker.

After one hour to remove OP50, 15 worms per well were moved from the S-Basal to the appropriate pre-warmed bacterial solutions using a p200 pipette. To prevent loss due to worms sticking to the pipette, glass pipette tips were used. These were made by cutting Pasteur pipettes approximately 5 cm from the tip and connected to the p200 using a short piece of rubber tubing. Glass pipettes were stored in 95% ethanol and flamed and rinsed in distilled water before use and between each bacterial solution condition.

Worms were moved to fresh bacterial solutions twice weekly at which point they were scored for movement. Any non-moving worms were pipetted onto a RT Nematode Growth Media (NGM) plate [61]. Those that did not respond to gentle prodding with a worm pick were scored as dead. Any responsive worms were returned to the experiment. FUDR was added for the first 2 weeks of the experiment and the plates were maintained on a slow rotating shaker at 20°C throughout. 60 worms were used for each treatment.

For experiments involving *eat-2* mutants the egg lays were done 20 hours before N2 wild type worms to ensure both groups were adults before FUDR was added.

### Pumping rates

Worms were reared as if entering a BDR lifespan study. After 24 hours in either a 1.5 OD or 0.15 OD bacterial solution they were transferred to 96 well cell culture plates containing 150 µl of the appropriate bacterial solution, one worm per well. After being left for 1 hour at 20°C on a shaker in the 96 well plates the number of pharyngeal pumps in a 30 second period was recorded for each worm. This was done twice for each worm.

### Direct feeding studies

*E. coli* (DH5α) carrying the topo cloning vector, pCR2.1, with tdTOMATO inserted between EcoRI sites was grown in 100 mls LB+carb at 37°C for 24 hrs. Bacterial solutions were then washed and prepared in the same manner described for BDR media above. N2 wild type worms were grown as described for BDR lifespan studies and transferred into either 1.5 OD or 0.15 OD fluorescent bacteria solutions. One 12-well plate was used and four wells with 15 worms per well were setup for each OD, resulting in 60 worms per condition. Worms were placed at 20°C on a “belly-dancer” for 24 hours. To remove external bacteria, worms were then pipetted into S-basal briefly and transferred into 10 µl sodium azide (20 mM) on an NGM plate. Five animals from 1.5 OD and 0.15 OD were positioned next to each other and images were taken using a Leica fluorescent dissecting scope and Leica LW4000 software. The process was repeated until 20 animals per OD were imaged. Photoshop CS3 was used to calculate mean fluorescent pixel intensity per worm. The entire process was repeated three days later with a new set of worms and bacteria to yield a replicate study.

### Growth rates

Developmental stage synchronized worms were grown on OP50 NGM plates until the L2 larval stage at which point they were moved to either a 1.5 OD or 0.15 OD bacterial solution. No FUDR was added. After 48 hours in liquid culture, sample worms were removed and photographed using a light microscope under azide anesthesia.

### Strain construction

Primers for genotyping the AAK mutants were as follows:

AAK1 oAD722 – 5′ external to deletion: TAGAGTTTCCCTTTCTTCGCTCAC
AAK1 oAD723 – 5′ internal to deletion: CATATTCAAACCGGATACGACGTC
AAK1 oAD742 – 3′ external to deletion: GCAACACTCTGAACCACATCAATATC
AAK2 oAD 743 – 5′ external to deletion: GATGTCGTTGGAAAGATTCGCC
AAK2 oAD 720 – 5′ internal to deletion: TCATGATTATGGAGCACGTTTCCG
AAK2 oAD744 – 3′ external to deletion: CAATGCTGAGGTGACTTCCTCTTCG

Cross to generate *aak-1(tm1944) III; aak-2(ok524) X* double mutant was performed between *aak-1(tm1944)* males and *aak-2(ok524)* hermaphrodites. See Figure S1a for genotype confirmation.

### Oxygen saturation

Oxygen saturation was compared between BDR media at 1.5 OD and 0.15 OD with and without worms. Two 12-well culture plates were set up for 1.5 OD and two plates for 0.15 OD using BDR media prepared as if for a BDR lifespan. N2 wild type worms reared as if entering BDR lifespans were placed, 15 worms per well, in the wells of one plate for each OD. Plates were rotated on a shaker at 20°C for 24 hours, at which point the BDR media from each plate was pooled into a 50 ml culture tube. Percent oxygen saturation in relation to air was measured using an oxygen probe. Oxygen saturation for each condition was measured twice more with a ten-minute period between each measurement.

### Reproduction studies

Worms were reared as if entering a BDR lifespan study but 72 hours after the egg lay adult worms were picked from the plates and transferred to 3 ml of either 1.5 OD or 0.15 OD pre-warmed bacterial solution and rotated on a shaker at 20°C for 2 hours. After 2 hours, worms were pipetted into individual wells of a 96 well plate containing 150 µl of the appropriate bacterial solution. Care was taken to avoid transferring any eggs. No FUDR was used for the reproduction studies. Worms were left in single wells for 7 hours at 20°C on the shaker before the plate was removed and left settle for 10 min. The top 100 µl of bacterial solution was removed from all wells to allow visualization of eggs in the high bacterial concentration soln. Tests verified this supernatant did not contain any eggs. Eggs settled at the bottom of well were then scored. Sample size was 48 worms per treatment.

### Phospho-AMPK levels

Antibody against phospho-AMPK (T172) was obtained from Cell Signaling Technology and Western blotted according to manufacturers instructions using lysates from mixed age nematode populations.

### Statistical Analysis

All statistics were performed using JMP statistic analysis software.

## Supporting Information

Figure S1 Construction of aak-1(tm1944) III; aak-2(ok524) X double mutants. a. 2 5′ Sequencing primers were designed (see methods) either external (Ex) or internal (In) to the deletion for aak-1(tm1944) (1) or aak-2(ok524) (2) along with a 3′ primer external to the deletion. Single worm PCR confirmed worms as being homozygous for both deletions as visualized by the presence of only a truncated PCR product when the 5′ external primer is used and no product using the internal 5′ primer. b. aak-1(tm1944) (1) or aak-2(ok524) double mutant worms (aak −/−) show no phospho-AMPK activity as determined by phospho-specific antibody western blot analysis. Tubulin acts as a loading control.
(4.33 MB TIF)

Table S1 Oxygen saturation in control and BDR media.
(0.03 MB DOC)
