# Supplementary figures and images for: Optimizing Dietary Restriction for Genetic Epistasis Analysis and Gene Discovery in C. elegans
Source: PLoS One. 2009 Feb 20;4(2):e4535. doi: 10.1371/journal.pone.0004535 (PMC2643252; doi:10.1371/journal.pone.0004535)

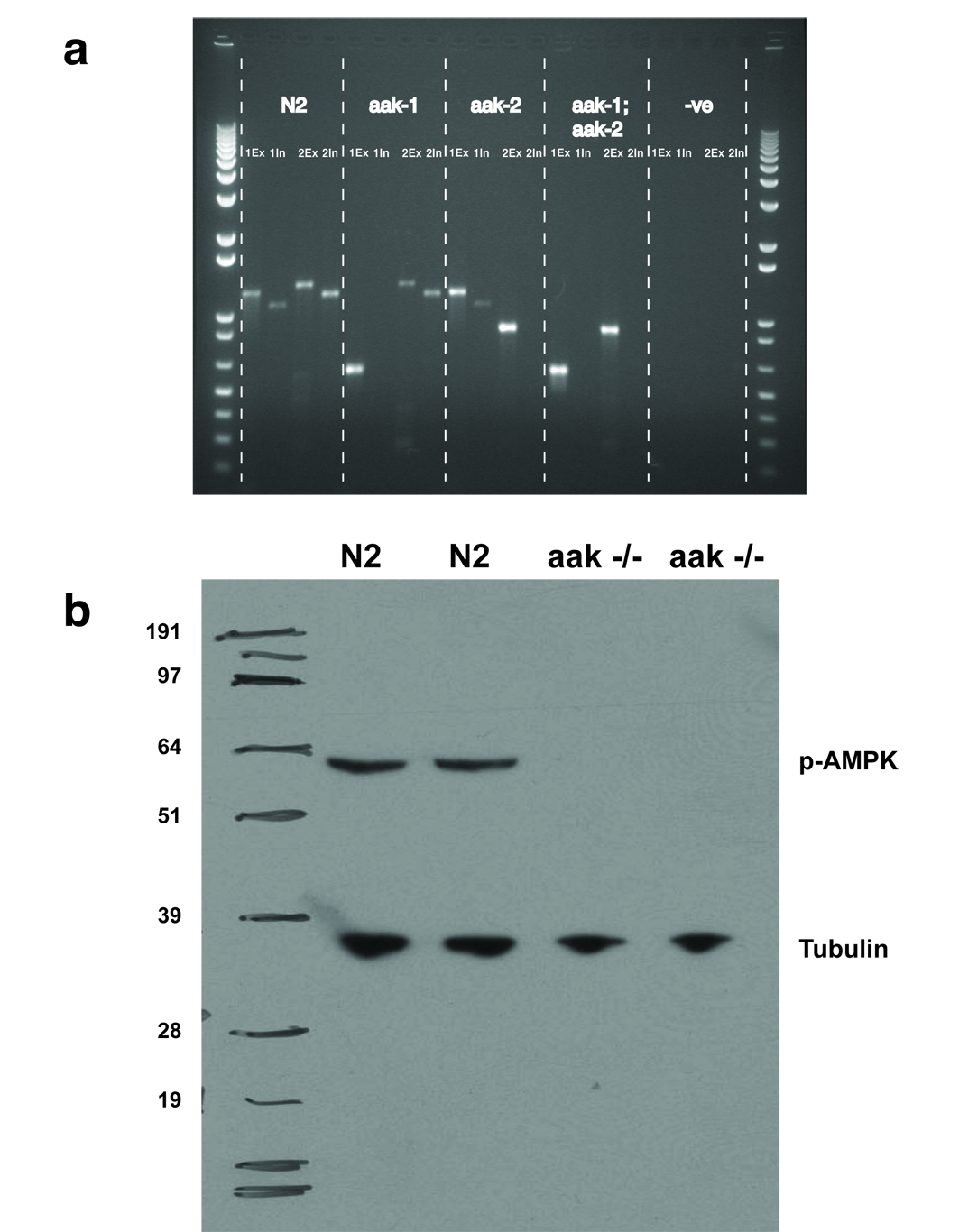

Supplement: Figure S1 — Construction of aak-1(tm1944) III; aak-2(ok524) X double mutants. a. 2 5′ Sequencing primers were designed (see methods) either external (Ex) or internal (In) to the deletion for aak-1(tm1944) (1) or aak-2(ok524) (2) along with a 3′ primer external to the deletion. Single worm PCR confirmed worms as being homozygous for both deletions as visualized by the presence of only a truncated PCR product when the 5′ external primer is used and no product using the internal 5′ primer. b. aak-1(tm1944) (1) or aak-2(ok524) double mutant worms (aak −/−) show no phospho-AMPK activity as determined by phospho-specific antibody western blot analysis. Tubulin acts as a loading control. (4.33 MB TIF) [file pone.0004535.s001.tif]
