# Supplementary material for: Optimizing Dietary Restriction for Genetic Epistasis Analysis and Gene Discovery in C. elegans
Source: PLoS One. 2009 Feb 20;4(2):e4535. doi: 10.1371/journal.pone.0004535 (PMC2643252; doi:10.1371/journal.pone.0004535)
Supplement: Table S1 — Oxygen saturation in control and BDR media. (0.03 MB DOC) [file pone.0004535.s002.doc]

**Supplementary Table 1. Oxygen saturation in control and BDR media.**

| **BDR Sample** | **Mean % Oxygen** | **Standard Deviation** | **% Oxygen Reading 1** | | **% Oxygen Reading 2** | | **% Oxygen Reading 3** |
| --- | --- | --- | --- | --- | --- | --- | --- |
| OD 1.5 with worms | 95.6% | 1.4 | 97.1% |  | 95.5% |  | 94.3% |
| OD 0.15 with worms | 96.1% | 0.45 | 95.6% |  | 96.5% |  | 96.1% |
| OD 1.5 without worms | 95.6% | 0.5 | 95.5% |  | 95.1% |  | 96.1% |
| OD 0.15 without worms | 95.7% | 0.46 | 95.3% |  | 96.2% |  | 95.6% |
